# An improved automated immunoassay for C-reactive protein on the Dimension^®^ clinical chemistry system

**DOI:** 10.1155/S1463924600000195

**Published:** 2000

**Authors:** Tie Q. Wei, Steve Kramer, Victor P. Chu, Dave Hudson, Daniel Kilgore, Sue Salyer, Grace Parker, Amy Eyberger, Rene Arentzen, Heikki Koiv

**Affiliations:** Dade Behring Glasgow Business Community PO Box 6101 Newark DE 19714-6101 USA

## Abstract

Recent clinical data indicate that the measurement of the concentration of C-reactive protein (CRP) requires a higher sensitivity and wider dynamic range than most of the current methods can offer. Our goal was to develop a totally automated and highly sensitive CRP assay with an extended range on the Dimension^®^  clinical chemistry system based on particle-enhanced turbidimetric-immunoassay (PETIA) technology. The improved method was optimized and compared to the Binding Site's radial immunodiffusion assay using disease state specimens to minimize interference. Assay performance was assessed on the Dimension^®^ system in a 12-instrument inter-laboratory comparison study. A split-sample comparison (n = 622) was performed between the improved CRP method on the Dimension^®^ system and the N Latex CRP mono method on the Behring Nephelometer, using a number of reagent and calibrator lots on multiple instruments. The method was also referenced to the standard material, CRM470, provided by the International Federation of Clinical Chemistry (IFCC). The improved CRP method was linear to 265.1mg/l with a detection limit between 0.2 and 0.5mg/l. The method detects antigen excess from the upper assay limit to 2000mg/l, thereby allowing users to retest the sample with dilution. Calibration was stable for 60 days. The within-run reproducibility (CV) was less than 5.1% and total reproducibility ranged from 1.1 to 6.7% between 3.3 and 265.4mg/l CRP. Linear regression analysis of the results on the improved Dimension^®^ method (DM) versus the Behring Nephelometer (BN) yielded the following equation: DM = 0.99 × BN − 0.37; r = 0.992. Minimal interference was observed from sera of patients with elevated IgM, IgG and IgA. The recovery of the IFCC standard was within 100 ± 7 % across multiple lots of reagent and calibrator. The improved CRP method provided a sensitive, accurate and rapid approach to quantify CRP in serum and plasma on the Dimension^®^ clinical chemistry system. The ability to detect antigen excess eliminated reporting falsely low results caused by the ‘prozone effect’.


Journal of Automated Methods & Management in Chemistry, Vol. 22, No. 5 (September–October 2000) pp. 125–131

### An improved automated immunoassay for C-reactive protein on the Dimension® clinical chemistry system

**Tie Q. Wei*, Steve Kramer, Victor P. Chu, Dave Hudson, Daniel Kilgore, Sue Salyer, Grace Parker, Amy Eyberger, Rene Arentzen and Heikki Koiv**

*Dade Behring, Glasgow Business Community, PO Box 6101, Newark, DE 19714-6101, USA*

*Recent clinical data indicate that the measurement of the concentration of C-reactive protein (CRP) requires a higher sensitivity and wider dynamic range than most of the current methods can offer. Our goal was to develop a totally automated and highly sensitive CRP assay with an extended range on the Dimension® clinical chemistry system based on particle-enhanced turbidimetric-immunoassay (PETIA) technology. The improved method was optimized and compared to the Binding Site's radial immunodiffusion assay using disease state specimens to minimize interference. Assay performance was assessed on the Dimension® system in a 12-instrument inter-laboratory comparison study. A split-sample comparison ($n = 622$) was performed between the improved CRP method on the Dimension® system and the N Latex CRP mono method on the Behring Nephelometer, using a number of reagent and calibrator lots on multiple instruments. The method was also referenced to the standard material, CRM470, provided by the International Federation of Clinical Chemistry (IFCC). The improved CRP method was linear to 265.1 mg/l with a detection limit between 0.2 and 0.5 mg/l. The method detects antigen excess from the upper assay limit to 2000 mg/l, thereby allowing users to retest the sample with dilution. Calibration was stable for 60 days. The within-run reproducibility (CV) was less than 5.1% and total reproducibility ranged from 1.1 to 6.7% between 3.3 and 265.4 mg/l CRP. Linear regression analysis of the results on the improved Dimension® method (DM) versus the Behring Nephelometer (BN) yielded the following equation: $DM = 0.99 \times BN - 0.37$; $r = 0.992$. Minimal interference was observed from sera of patients with elevated IgM, IgG and IgA. The recovery of the IFCC standard was within $100 \pm 7\%$ across multiple lots of reagent and calibrator. The improved CRP method provided a sensitive, accurate and rapid approach to quantify CRP in serum and plasma on the Dimension® clinical chemistry system. The ability to detect antigen excess eliminated reporting falsely low results caused by the 'prozone effect'.*

### Introduction

C-reactive protein (CRP) is an acute phase reactant that has the ability to activate complement after binding to antigen, and in combination with macrophages to kill bacteria and tumour cells [1–3]. The concentration of CRP in plasma increases in response to a variety of acute or chronic stimuli. These stimuli include infection, inflammation, trauma, surgery, neoplasia or tissue destruction [4, 5]. Recently, a number of articles reported the associations of serum CRP concentration with cardiac risk, unstable angina, myocardial infarction and recurrent coronary events [6–17]. As these more recent clinical utilities of CRP assays have been revealed, the upper reference limit has been decreasing. Although the current consensus reference range has already decreased from < 8.2 mg/l to < 5 mg/l [4], clinical data showed that less than 3 mg/l CRP is desirable [17]. CRP values greater than 3 mg/l may indicate a greater risk of having cardiovascular disease, e.g. unstable angina and myocardial infarction. A recent study suggested that CRP levels even at less than 3 mg/l might help predict the relative risks of the first myocardial infarction when stratified by lipoprotein levels and smoking history [8]. This trend toward a decreased upper reference limit challenges the analytical sensitivity of most current CRP methods on automated analysers. For example, the Abbott® TDx® method claims 12% CV at 10 mg/l and the Beckman® Array® method has a detection limit of 4 mg/l. The existing CRP method on Dimension® systems has a detection limit of 2 mg/l and ~15% CV at 4 mg/l. Therefore, none of these methods is sensitive enough for accurate and precise measurement of CRP at 3–5 mg/l.

On the other hand, new clinical findings also call for extending the upper assay limit for the CRP assay. A CRP value of 120, 140 or 150 mg/l has been used as the cutoff level for necrotizing pancreatitis in several clinical studies [18–22]. A peak concentration of 74–166 mg/l CRP after acute myocardial infarction was suggested for prognosis of mortality [12]. In patients who died due to congestive heart failure, the mean peak serum CRP concentration was 226 mg/l. The mean CRP concentration in those who suffered sudden cardiac death was 167 mg/l. The existing CRP assay (up to ~100 mg/l IFCC standardized value) on the Dimension® system cannot meet the demands for the higher upper assay limit. In addition, the frequency of high CRP concentration in patient samples makes detection of antigen excess (prozone effect) a necessity that the current Dimension® CRP assay is unable to deliver.

This paper describes a new improved particle-enhanced turbidimetric CRP assay on the Dimension® system that offers the high analytical sensitivity and extended upper assay limit required for a broad range of clinical applications. In addition, the new assay also has the ability to signal antigen excess up to 2000 mg/l CRP. The assay principle and performance are detailed in this article.

* e-mail: weitq@dadebehring.com

### Materials and methods

The improved CRP (RCRP) Flex™ reagent cartridges were from Dade Behring. These contained particle reagent and buffer. As does the existing Dimension® CRP method, this improved CRP assay uses particle-enhanced turbidimetric immunoassay technology (PETIA). Latex particles, to which goat anti-CRP polyclonal antibodies are covalently attached, aggregate in the presence of CRP. The rate of increase of turbidity caused by particle aggregation is measured bichromatically at 340 and 700 nm. The antibody used in the particle reagent is the ion exchange-purified IgG fraction of a goat polyclonal anti human-CRP antibody obtained from Consolidated Technology, TX. The antibody is covalently coupled to 48 nm chloromethylstyrene and polyvinylnaphthalene particles. The antibody is loaded at 2–2.2 mg/ml in a particle solution that contains 0.45% solids in a 15 mM sodium phosphate coupling buffer, pH 7.5. The buffer contains 245 mM potassium phosphate and 1.88% polyethylene glycol.

Specimens (serum, plasma) were from several hospitals. Dade® Liquid Immunology Control, Biorad® Liquichek™ Immunology Control, Biorad® Lyphochek™ Immunology Control, Chiron® Immunology Control, Dade Behring® Rheumatology Control, National Institute for Biological Standards, Dade® Liquid Immunology Control and Beckman Vigil® plus control were used as quality control materials. For recovery and linearity studies, the standard material from International Federation of Clinical Chemistry (IFCC), CRM 470 [30, 31] was hydrated according to instructions to give the reported concentration of 39.2 mg/l. For stability studies, CRP calibrator containing CRP concentrations of ~0.0, 20.0, 38.0, 120.0 and 260.0 mg/l was used. Calibration was performed with Dimension® system RCRP calibrator (Dade Behring). Purified CRP stock solution was purchased from SCIPAC (SCIPAC, Sittingbourne, UK) and was used for making the master pool and calibrator. The calibrator bottle values were assigned with a master pool that was anchored to the IFCC standard, CRM 470 [30, 31], using a number of Dimension® instruments.

The CRP stock purchased from SCIPAC was also used in the interference study. Both the interference test compound and the CRP stock were added into a low CRP human serum pool to obtain the desired concentrations. The test results were compared to the results of controls that were made by adding the same volume of the compound and CRP-free solutions.

Radial immuno-diffusion assays were performed using the Binding Site's CRP kit (Lot No. GA044, the Binding Site, UK). A standard curve was generated using dilutions of a high level calibrator included in the kit. The calibrator was diluted in water to concentrations of 52, 31.2, 5.2 and 0.156 mg/l. Ring diameters were measured using a jeweller's eyepiece. Two RID plates were used per sample. The standard curve was generated on plate one, the other plate contained the high level calibrator only. All plates contained a control serum sample at 29.6 mg/l that was included in the kit. Standards and samples were loaded into respective plate wells and incubated in a room temperature incubator (23–27°C) for 72 h. Ring diameters were measured using a Bio-Rad's immunodiffusion reader. A ring diameter ($mm^2$) versus CRP concentration (mg/l) curve was generated and sample concentrations determined.

A split-sample correlation study was performed in laboratories of the authors and two clinical hospital laboratories. Serum and plasma specimens used in the author's laboratories were acquired from several hospital laboratories, shipped on dry ice, stored frozen at $-20$°C and thawed before use. Clinical specimens used in hospital laboratories were either fresh or treated the same way. Analyses were performed on both the Dimension® system and Behring Nephelometer® analysers (BNII or BNA) on the same day. Passing and Bablok regression [25, 26] was performed using the MedCalc® software package purchased from MedCalc, Belgium.

The inter-laboratory comparison (ILC) was performed with a full-factorial design, in which individual instruments and days of the study were the experimental factors. BioRad® Liquichek™ Immunology controls, two serum pools and one lot of calibrator were run in five replicates per day over five consecutive days on each of 12 calibrated Dimension® systems, including the RxL, XL and AR models. The instruments were physically located in a number of separate laboratories. We used JMP® statistical software (SAS Institute) to analyse the data.

Processing of the improved CRP assays on the Dimension® system, as directed by the system software is depicted and described briefly in figure 1. Data shown there were captured in a non-routine processing mode, in which absorbances are monitored continuously. The particle aggregation in the presence of CRP is quantified by subtracting bichromatic R2 absorbance from that for R3 as shown in the figure, the difference being used with the calibration coefficients for the analyte computation. Operating principles of the Dimension® system have been previously published [23].

### Results and discussion

To achieve better linearity at low CRP concentrations, the rate of absorbance change was converted to a transformed rate based upon an empirical mathematical model. Shown in figure 2 is a typical standard curve with the transformed rate, which indicates agglutination sufficient for application in a clinical assay. The particle reagent formulation was optimized by adjusting the concentration, size and antibody loading of the solid particles; the formulation was selected for precise analytical results across the range of the standard curve. The curve shape and the overall range of the transformed absorbance changes for the optimized reagents are shown in figure 2. This rate transformation helped in achieving a linear CRP method at low analyte concentrations.

To ensure this method can be utilized in a wide variety of clinical conditions, we tested the effects of physiological and disease state substances on the results in two ways.

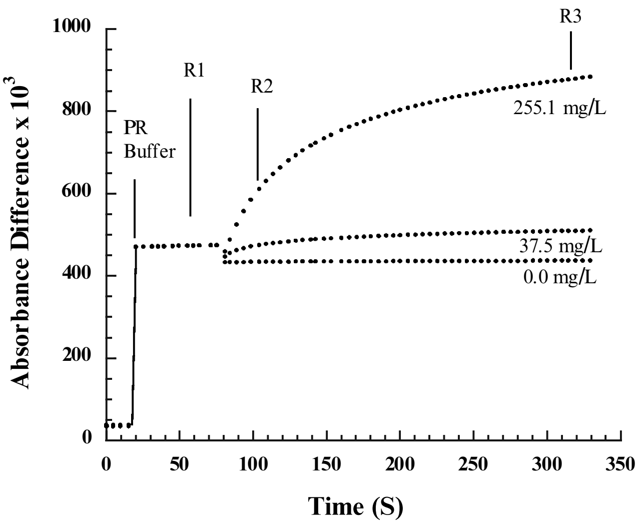

*Figure 1. Absorbance change over time in the improved CRP assay. Absorbance was measured continuously (non-routine mode of instrument operation) on a Dimension® system model AR at 340 and 700 nm wavelengths. R1, R2 and R3 indicate the times at which the instrument measures these absorbances during routine assay processing. The lines show the measured differences in the two absorbances over time for 0.0, 37.5 and 255.1 mg/l CRP calibrator. In the routine operating mode, particle reagent (PR) and buffer are first added to the cuvette, chased by water, and mixed ultrasonically. Next, 10 μl of sample is added, chased by water, and the contents ultrasonically mixed. R1 measurement is performed to detect any unusual reagent delivery which would be flagged as an error. Measurements R2 and R3 are made at a fixed time. The measured bichromatic R2 absorbance is subtracted from that for R3, the difference being proportional to the concentration of CRP in the sample.*

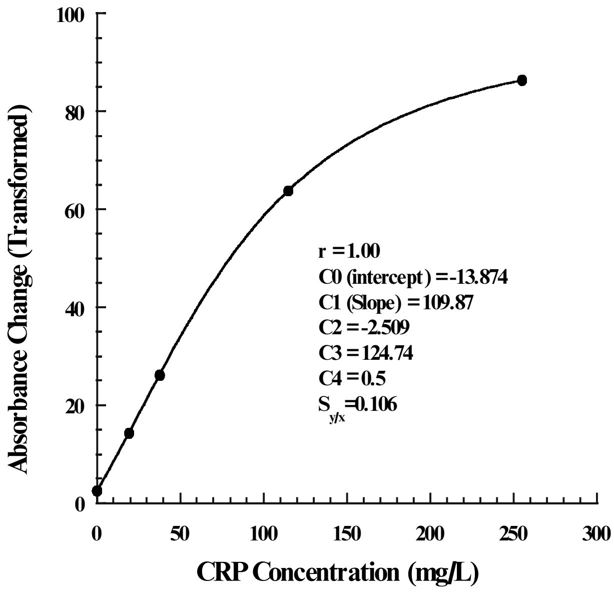

*Figure 2. Representative CRP calibration curve. Data were obtained on a Dimension® system, model AR. Points show means of duplicate determinations for calibrators with five concentrations of CRP. The logit curve fit was used to obtain calibration coefficients for the slope, intercept, C2, and C3 with a fixed C4 term* (= 0.5).

*Table 1. Performance of the improved CRP assay on Dimension® system–interference from physiological substances.*

| *Physiological substance*[a] | *32–38 mg/l CRP error (%)* | *110–140 mg/l CRP error (%)* |
|---|---|---|
| Haemoglobin (1000 mg/dl) | −2.0 | −4.9 |
| Conjugated bilirubin (60 mg/dl) | −1.4 | −4.3 |
| Unconjugated bilirubin (500 mg/dl) | 2.1 | −0.3 |
| Cholesterol (500 mg/dl) | 2.6 | 0.6 |
| Triglycerides (800 mg/dl) | 7.4 | 3.4 |
| Albumin (6.8 g/dl)[b] | −5.4 | −1.9 |
| Total protein (11.8 g/dl)[b] | −4.0 | −2.6 |
| Total protein (3.5 g/dl)[b] | 3.0 | −3.6 |
| Rheumatoid factor (571 IU/ml) | 8.3 | 5.6 |

[a]Unless noted otherwise, the test substance was added to a low CRP normal human serum pool along with CRP stock solution to give the desired concentrations. The control was the same pool with no added test compound but the same amount of CRP present.

[b] The serum pool containing the stated concentrations of the test compound was spiked with the CRP stock solution to give the desired CRP concentrations. The control was the normal human serum with the same amount of CRP present.

Error = [(observed result − control result)/control result] × 100.

First, we studied the effect of the added substances that are often encountered clinically and may potentially interfere with the assay. For instance, because the CRP assay is widely used in neonatal care, we tested the improved CRP assay for interference with unconjugated bilirubin (table 1). That no significant interference was found with 60 mg/dl of unconjugated bilirubin indicates that this assay may be used safely for patients who have developed severe jaundice. The specificity characteristics of the assay in the presence of other physiological substances are also indicated in table 1. No significant ($< 10\%$) interference was found in an extensive interference study using 34 other drugs or compounds.

Second, we compared the CRP results of disease state specimens measured by this assay to those obtained using the radial immuno-diffusion assay. Because the radial immuno-diffusion assay does not use latex particles, it is free of the interference caused by non-specific agglutination seen in regular latex particle assay. It was reported that specimens of a myeloma patient with elevated IgM interfered with a commercial CRP assay using anti-CRP antibody-coated latex particles [24]. A myeloma patient sample (IgM 60.0 g/l) was tested and the commercial assay reported a CRP value of 274 mg/l while a radial immuno-diffusion method (RID) that uses the same anti-CRP antibody reported only 6 mg/l. The authors concluded that IgM from the patient might have bound to the latex particles coated with anti-CRP antibody and caused non-specific particle agglutination, which in turn resulted in falsely elevated results. To test if elevated IgM interfered with our method, we measured the CRP values of the sera of two myeloma patients containing elevated IgM using both the improved CRP method and an RID assay obtained from the Binding Site, UK. The serum

*Table 2. Comparison of the CRP results of myeloma patients measured with the Binding Site radial-immunodiffusion assay and the improved CRP method on Dimension error® system.*

| *Myeloma sample* | *Paraprotein (g/l)* | *RID result (mg/l)* | *Dimension® result (mg/l)* |
|---|---|---|---|
| 1 | IgM (26.1) | 0.0 | −0.1 |
| 2 | IgM (23.7) | 59.4 | 59.9 |
| 3 | IgG (43.4) | 156.0 | 146.2 |
| 4 | IgG (56.3) | 3.1 | 4.0 |
| 5 | IgA (6.2) | 10.6 | 8.4 |
| 6 | IgA (22.7) | 45.9 | 42.3 |

Each Dimension® result was measured in duplicate while each RID result was obtained by a single determination.

*Table 3. Reproducibility analysis of serum and commercial control samples.*†

| *Sample* | *Observed mean (mg/l)* | *Within-run SD (CV%) (mg/l)* | *Total SD (CV%) (mg/l)* |
|---|---|---|---|
| Serum pool 1 | 3.3 | 0.2 (4.8) | 0.2 (6.7) |
| Serum pool 2 | 39.0 | 0.6 (1.5) | 0.8 (2.0) |
| Serum pool 3 | 260.8 | 0.43 (1.7) | 0.56 (2.1) |
| Biorad® Lyphochek™ Level 1 | 6.4 | 0.1 (0.9) | 0.2 (2.4) |
| Biorad® Liquichek™ Level 1 | 13.3 | 0.1 (0.9) | 0.2 (1.8) |
| Biorad® Liquichek™ Level 2 | 30.0 | 0.1 (0.4) | 0.6 (2.0) |

† Measurements were performed on a Dimension® system model XL. NCCLS protocol EP5-T2 was followed with each sample run in duplicate twice a day for 20 days.

IgM values of the two patients are 26.1 and 23.7 g/l, respectively. The corresponding CRP values reported with the RID method were 0.0 and 59.4 mg/l, respectively, as compared to − 0.1 and 59.9 mg/l measured by the improved CRP assay (table 2). This observation indicates the myeloma sera containing elevated IgM did not interfere with the improved Dimension® CRP assay. In addition, sera from myeloma patients containing elevated IgG or IgA were also tested by the improved assay and the RID method, no significant interference was found in these studies (table 2). Icteric, lipemic and haemolysed sera from patients were also tested using both methods, no significant difference in the measured CRP values was detected (data not shown).

Both within-run and total precisions were excellent across the assay range, as summarized in table 3. The data were obtained using a Dimension® system (model XL), but are representatives of the precision observed for all of the three instrument models used in this testing (AR, XL and RxL). While precision on individual instruments provides important information about the assay, it does not indicate the total method variability such as might be observed in a multi-site proficiency survey. We thus performed an inter-laboratory comparison (ILC) study as described above. The results are reported in table 4. The overall standard deviations, which may be taken as a realistic predictor of the variability expected in multi-site surveys, indicate very good multi-laboratory performance with one reagent and one calibrator lot.

Like other direct agglutination assays, for a given amount of antibody particles, the particle–antigen complex formation increases with the amount of CRP to a point beyond which there is less complex formed. This phenomenon of less complex formation with increasing amounts of antigen indicates antigen excess and is called the 'hook effect' or 'prozone effect'. This assay started to show antigen excess between 360 and 400 mg/l (figure 5). However, a software routine was incorporated into the method parameters to identify and signal the antigen excess situation. Samples with CRP concentrations either above the assay range (250.0 mg/l) or in antigen excess situations trigger an error message (either 'assay range' or 'antigen excess', respectively). This allows operators to retest the sample by dilution. Figure 5 shows the signal over analyte concentrations spanning the range from 0.0 to 2000.0 mg/l CRP. Any sample between the upper assay limit (250.0 mg/l) and 2000 mg/l was flagged. Theoretically, even with samples above 2000 mg/l, the method should be able to flag antigen excess, but it was not tested for all the reagent lots manufactured.

The results of a split-sample method comparison study, shown in figure 3 for the subject assay in comparison to the Behring Nephelometric analyser, demonstrated very good correlation. The patient samples used in this study include serum, plasma and disease state specimens, e.g. icteric, haemolysed and lipemic samples as well as myeloma specimens with elevated IgG, IgA and IgM. For maximum robustness of the comparisons investigated, we used a number of reagent lots and multiple instruments

*Table 4. Inter-laboratory comparison study for the improved CRP method on the Dimension® system.*†

| *Samples* | *Measured mean (mg/l)* | *Overall SD (mg/l)* | *Overall CV (%)* |
|---|---|---|---|
| Calibrators (bottle value in mg/l) | | | |
| Level 1 (0.0) | 0.1 | 0.2 | — |
| Level 2 (2.0) | 19.8 | 0.8 | 3.9 |
| Level 3 (38.9) | 39.8 | 1.6 | 3.9 |
| Level 4 (125.4) | 126.8 | 4.9 | 3.8 |
| Level 5 (265.7) | 268.9 | 9.6 | 3.6 |
| Serum pools | | | |
| Pool 1 | 3.3 | 0.2 | 5.0 |
| Pool 2 | 28.4 | 0.9 | 3.3 |
| BioRad Liquichek Control (target ± range) | | | |
| Level 1 (15.4 ± 3.0) | 14.4 | 0.4 | 2.6 |
| Level 2 (25.7 ± 5.2) | 24.7 | 0.5 | 2.0 |
| Level 3 (36.0 ± 7.2) | 35.6 | 0.9 | 2.5 |

† All samples were measured in five replicates per day for 5 days using 12 Dimension® instruments.

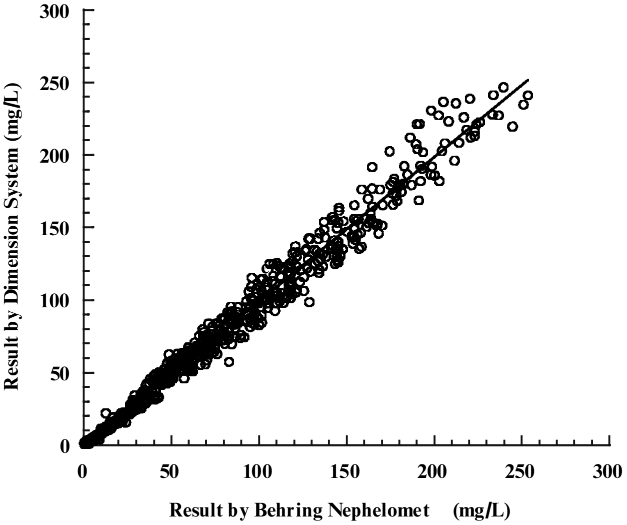

*Figure 3. Comparison of CRP results as reported on the Dimension® system with results from the Behring Nephelometer (N Latex CRP Mono) assay. The data represent duplicate determinations for 622 clinical specimens. Each set of duplicates was performed on one of three Dimension® systems used for the complete study (one in the laboratories of the authors and two in separate clinical hospital laboratories). Three Behring Nephelometer analysers, two BNII and one BNA model, were employed during the study. Two reagent lots and two calibrator lots were used for the Dimension® system, and one standard lot with four reagent lots were used on the Behring Nephelometer analysers. Values for the regression line are as follows:* $r = 0.992$; $n = 622$; $S_{y/x} = 7.04$ *mg/l; slope* $= 0.993 \pm 0.004$; *intercept* $= -0.374 \pm 0.402$ *mg/l.*

and calibrators in the study, as detailed in the caption of figure 3. The entire study occurred during a 6-month period.

We also tested the same set of data with Passing and Bablok regression [25, 26]. The advantage of using Passing and Bablok regression is the elimination of the effect of extreme points that could be over weighted by standard linear regression. The regression statistics of Passing and Bablok for the correlation between the improved CRP (DM) and the Behring Nephelometric method (BN) are: DM = 0.984 × BN − 0.033 (mg/l). The slope (95% confidence interval: 0.975–0.993) given here is similar to that obtained using linear regression (0.993 ± 0.004) as shown in figure 3. However, the intercept is closer to zero (95% confidence interval: − 0.326 to 0.197 mg/l) after eliminating the effect of extreme data points, as compared to the intercept obtained from the linear regression (− 0.776 to 0.028 mg/l).

The Bland–Altman form of the difference plot [27, 28] is also provided in figure 4 to show the measure of agreement between the two methods. It is apparent that there is no obvious relationship between the differences and measured concentrations. The mean difference and the standard error of the mean differences (SEM) were calculated to be − 0.8 and 0.265 mg/l, respectively. The 95% confidence interval for the mean difference (estimated as the mean ± 2 × SEM) was − 0.3 and − 1.36 mg/l. Although this confidence interval does not include 0.0 mg/l, it is at a level comparable to the sensitivity of the assay (0.2–0.5 mg/l), and therefore indicates that bias between the Behring Nephelometric method and the improved assay is negligible.

A direct comparison of serum with plasma results was performed on specimens to which CRP had been added. This approach was used to demonstrate the equivalence of the two sample matrices because of the lack of available matched draws from patients with adequate CRP concentrations to span the assay range. This study, which included the anticoagulant sodium EDTA and lithium heparin showed the equivalence of the two specimen types and serum. The linear regression statistics obtained were sodium EDTA result = 1.01 × serum result + 0.7 (mg/l, $n = 45$), and lithium heparin result = 0.98 × serum result − 0.3 (mg/l, $n = 53$). Actual patient plasma specimens containing CRP, when compared using the Dimension® system and the Behring Nephelometer analyser gave correlation slopes not statistically different from the correlation with serum specimens.

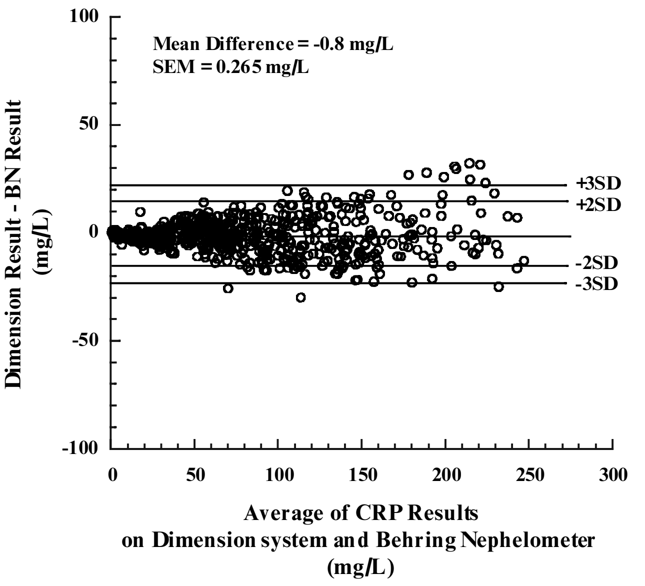

*Figure 4. Difference plot of the data used in figure 3 with the mean difference (bold line) and SD of the mean difference (thin line). Both two SD and three SD lines are shown in the graph. SEM represents the standard error of the mean difference.*

The accuracy of the method was further evaluated by recovery of the international standard, CRM 470. By the addition technique we found within 100 ± 7% recovery for three Flex™ reagent and calibrator lots.

The limit of detection was determined to be between 0.2 and 0.5 mg/l when defined as the concentration corresponding to two standard deviations above the 0.0 mg/l level ($n = 20$). This range was determined using four Flex™ reagent lots on six Dimension® RxL instruments conducted at three external clinical sites and the author's laboratory. Reproducibility studies performed with the 0.0 mg/l level using NCCLS protocol EP5-T2 showed a total SD of less than 0.2 mg/l with multiple instruments and reagent lots, and thus supported the results for the limit of detection.

Linearity was assessed by fitting the data to a quadratic model and by testing significance of the coefficient of the second-degree term [29]. This analysis indicated that the assay's linearity extended beyond 260 mg/l. Although linearity across the entire assay range is important, a more sensitive CRP method, which can be used to detect CRP at concentrations below the normal reference interval, must provide good linearity at low levels. Figure. 6 shows a dilution study performed with a serum sample diluted to 0.2 mg/l CRP with phosphate-buffered saline. The results indicate linearity sufficient for the measurement of CRP below the consensus cut-off level of 3–5 mg/l [4, 14].

Shelf life and calibration intervals are also important performance criteria for a clinical assay. Flex™ reagent cartridges, calibrated and periodically measured over 90 days, showed a maximum rate of change of 5% over a 60-day period of testing when tested with the upper four levels of calibrator. The zero-concentration calibrator showed no drift outside the limit of detection (0.5 mg/l). Based on this, a 60-day calibration interval was assigned. In continuing studies extended over 1 year using a 60-day calibration interval, the overall drifts for all calibrator levels were less than 5% at each calibrator level, thus a shelf life of at least 12 months was determined for this method.

A detailed comparison of the performance characteristics of the improved assay and the current CRP method on the Dimension® system is shown in table 5. Compared to the current commercial CRP assay, the improved method is five–10 times more sensitive and has ~2.5 times the assay range. It is also equipped with extra features, e.g. standardization with the IFCC reference CRM 470 [30, 31], antigen excess detection and faster throughput on certain instrument models (table 5).

In conclusion, this new assay offers more sensitive, precise and accurate CRP measurements than most other commercially available assays can deliver. The advantages make this improved assay suitable for a wide variety of clinical applications on this clinical chemistry system. The extended upper assay limit also decreases the need for re-testing of post-diluted samples and provides a faster turn-around time and lower cost per reportable result that is coupled with the improved throughput. We believe the addition of this improved CRP assay enhances the utility of the Dimension® system in laboratory settings where workstation consolidation is advantageous.

*Table 5. Performance characteristics of the improved CRP assay as compared to those of the current CRP method on the Dimension® system.*†

| *Characteristics* | *The improved CRP assay (RCRP)* | *CRP assay (CRP)* |
|---|---|---|
| Limit of detection | 0.2–0.5 mg/l | 1.7–2.0 mg/l |
| Upper assay limit | 250.0 mg/l | 100 mg/l |
| Detection of antigen excess | Yes | No§ |
| Throughput (batch mode on XL and RxL models) | 250 tests/h | 196 tests/h |
| Method comparison‡ (mg/l) | RCRP = 1.01 × CRP − 0.13; $n = 46$; $r = 0.998$; $S_{y/x} = 1.56$ | |

† CRP values shown here are the values anchored to the IFCC standard CRM 470 [27].

‡ Performed using linear regression. Passing and Bablok regression was also tested with the same data and gave a slope of 0.98 and an intercept of 0.07 mg/l.

§ The current commercial Dimension® CRP method demonstrated resistance to antigen excess up to 800 mg/l.

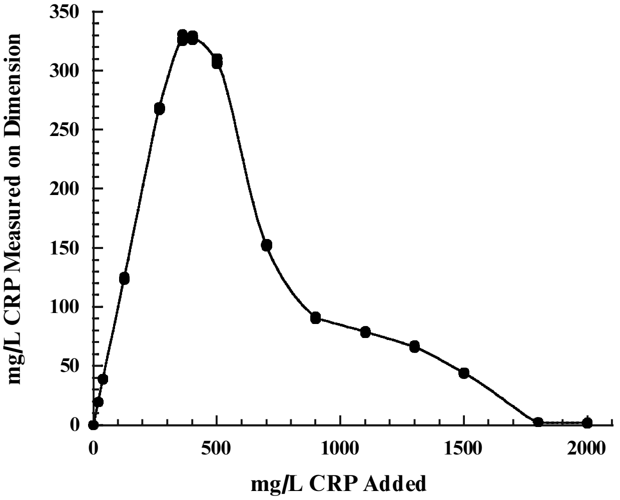

*Figure 5. Representative change of reaction rate with increasing CRP concentration demonstrating the antigen excess phenomenon of the improved CRP method. The antigen excess flag was validated using two reagent lots on 12 instruments. Samples with CRP values between the upper assay limit and 2000 mg/l triggered either the 'assay range' or 'antigen excess' flag. This routine assures that no falsely low CRP values are reported due to antigen excess and allows the operator to dilute and retest the sample.*

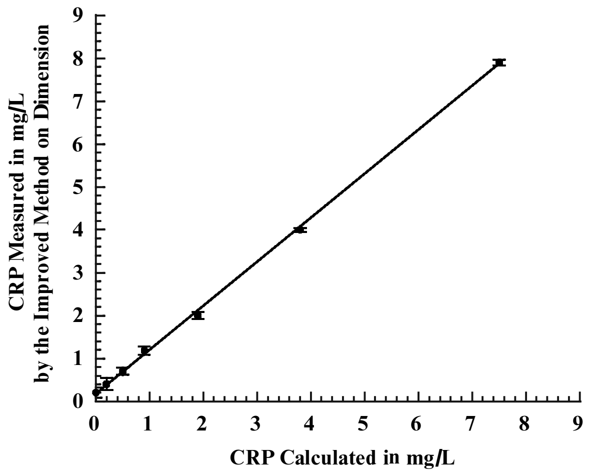

*Figure 6. A dilution study showing linearity at low CRP concentrations with the improved CRP assay. The sample was diluted with phosphate-buffer saline. Each data point represents the mean result of three independent determinations. The samples were tested in triplicate on a Dimension® AR system during each determination. The linear regression statistics are: measured = 1.03 × calculated = 0.14 (mg/l), r = 1.0. The error bars indicate mean ± one standard deviation.*

**Acknowledgements**

The authors thank Linda Duffy for her assistance in manuscript preparation.

Abbott® and TDx® are registered trademarks of Abbott Laboratories.

Biorad®, Biorad™ and Liquichek™, are trademarks of Biorad Laboratories.

Dimension® is a registered trademark of Dade Behring Inc.

Behring Nephelometer® is a registered trademark of Dade Behring Inc.

### References

1. Gambino, R., *Clin Chem.* **43** (1997), 2017.
2. Shrive, A. K., Cheetham, G. M. T., Holden, D, Myles, D. A. A., Turnell, W. G., Volanakis, J. E., Pepys, M. B., Bloomer, A. C. and Greenhough, T. J., *Nature Structural Biology*, **3** (1996), 346.
3. Kind, C. R. H. and Pepys, M. B., *Int. Med.*, **5** (1984), 112.
4. Tietz, N.W., *Textbook of Clinical Chemistry* (Philadelphia, PA: W. B. Saunders) (1999), 42–72 (specimen collection and processing), 481 (Interim Consensus Reference Intervals for 14 Plasma Proteins in Human serum Referenced to CRM 470/RPPHS), 713.
5. Gambino, R., C-reactive protein – an underutilized test. Lab Report for Physicians (1989), 11:41.
6. Rider, P. M., Rife, N., Pfeiffer, M. A., Sacks, F. M., Moue, L. A., Goldman, S., Flakier, G. C., *et al.*, *Circulation*, **98** (1998), 839.
7. Liuzzo, G., Buffon, A., Biasucci, L. M., Callimore, J. R., Caligiuri, G., Vitelli, A., Altamura, S., *et al.*, *Circulation*, **98** (1998), 2370.
8. Ridker, P. M., Glynn, R. J. and Hennekens, C. H., *Circulation*, **97** (1998), 2007.
9. Ridker, P. M., Cushman, M., Stampfer, M. J., Tracy, R. P. and Hennekens, C. H., *Circulation*, **97** (1998), 425.
10. Morrow, D., Rifai, N., Antman, E. M., Weiner, D. L., McCabe, C. H., Cannon, C. P. and Braunwald, E., *J. Am. Coll. Cardiol.*, **31** (1998), 1460.
11. Feld, R. D., *J. Clin. Ligand Assay*, **20** (1997), 313.
12. Pietila, K. O., Harmoinen, A. P., Jokiniitty, J. and Pasternack, A. I., *Eur. Heart J.*, **17** (1996), 1345.
13. Ueda, S., Ikeda, U., Yamamoto, K., *et al.*, *Am. Heart J.*, **131** (1996), 857.
14. Ridker, P. M., Cushman, M., Stampfer, M. J., Tracy, R. P. and Hennekens, C. H., *N. Engl. J. Med.*, **336** (1997), 973.
15. Haverkate, F., Thompson, S. G., Pyke, S. D. M., *et al.*, *Lancet*, **349** (1997), 462.
16. Maseri, A., *N. Engl. J. Med.*, **336** (1997), 1014.
17. Macy, E. M., Hayes, T. E. and Tracy, R. P., *Clin. Chem.*, **43** (1997), 52.
18. Chen, C. C., Wang, S. S., Lee, F. Y., Change, F. Y. and Lee, S. D., *Am. J. Gastroenterol.*, **94** (1999), 213.
19. Rau, B., Cebulla, M., Uhl, W., Schoenberg, M. H. and Beger, H. G., *Pancreas*, **17** (1998), 134.
20. Pezzilli, R., Billi, P., Miniero, R., Fiocchi, M., Cappelletti, O., Morselli-Labate, A. M., Barakat, B., Sprovieri, G. and Miglioli, M., *Dig. Dis. Sci.*, **40** (1995), 2341.
21. Imrie, C. W., *Schweiz Med. Wochenschr.*, **127** (1997), 798.
22. Rau, B., Pralle, U., Uhl, W., Schoenberg, M. H. and Berger, H. G., *J. Am. Coll. Surg.*, **181** (1995), 279.
23. Knight, D., Singer, R., White, J. M. and Fraser, C. G., *Clin. Chem.*, **34** (1988), 1899.
24. Yamada, K., Yagihashi, A., Ishii, S., Tanemura, K., Kida, T., Watanabe, N. and Niitsu, Y., *Clin. Chem.* **43**, 2435.
25. Passing, H. and Boblok, W., *J. Clin. Chem. Clin. Biochem.*, **21** (1983), 709.
26. Feldmann, U., Schneider, B., Klinkers, H. and Haeckel, R., *J. Clin. Chem. Clin. Biochem.*, **19** (1981), 121.
27. Bland, J. M. and Altman, D. G., *Lancet*, **1** (1986), 307.
28. Peterson, P. H., Stockl, D., Blaabjerg, O., Pedersen, B., Birkenose, E., Thienpont, L., *et al.*, *Clin. Chem.*, **43** (1997), 2039.
29. Burnett, R. W., *Clin. Chem.*, **26** (1980), 44.
30. Whicher, J. T., Ritchie, R. F., Johnson, A. M., Baudner, S., Bienvenu, J., Blirup-Jensen, S., Carlstrom, A., Dati, F., Ward, A. M. and Svendsen, P. J., *Clin. Chem.*, **40** (1994), 934.
31. Whicher, J. T., *Clin. Biochem.*, **31** (1998), 459.

